# Hepatic enzymes and immunoinflammatory response to Bio-C Temp bioceramic intracanal medication implanted into the subcutaneous tissue of rats

**DOI:** 10.1038/s41598-022-06545-5

**Published:** 2022-02-18

**Authors:** Camila Soares Lopes, Mateus Machado Delfino, Mário Tanomaru-Filho, Estela Sasso-Cerri, Juliane Maria Guerreiro-Tanomaru, Paulo Sérgio Cerri

**Affiliations:** 1 Department of Restorative Dentistry, Dental School, São Paulo State University (UNESP), Araraquara, SP Brazil; 2 Laboratory of Histology and Embryology, Department of Morphology, Genetics, Orthodontics and Pediatric Dentistry, Dental School, São Paulo State University (UNESP), Araraquara, SP Brazil

**Keywords:** Dental materials, Dental treatments, Endodontics

## Abstract

Our purpose was to evaluate the biocompatibility and hepatotoxicity of a new bioceramic intracanal medicament, Bio-C Temp (BIO). The biological properties of BIO were compared with calcium hydroxide-based intracanal medicament (Calen; CAL), used as gold pattern. Polyethylene tubes filled with BIO or CAL, and empty tubes (control group, CG) were implanted into subcutaneous tissue of rats. After 7, 15, 30 and 60 days, the samples were embedded in paraffin for morphological, quantitative and immunohistochemistry analyses. At 7 and 60 days, blood samples were collected for analysis of serum glutamic-oxaloacetic transaminase (GOT) and glutamic-pyruvic transaminase (GPT) levels. The data were submitted to two-way ANOVA and Tukey’s test (p ≤ 0.05). No significant difference was detected in serum GOT and GPT levels among BIO, CAL and CG specimens. In all periods, BIO specimens exhibited lower number of inflammatory cells and immunoexpression of IL-6, a pro-inflammatory cytokine, than CAL specimens. The reduction of these parameters was accompanied by significant increase in the collagen content and in the immunoexpression of IL-10, a cytokine involved in the tissue repair, over time. Our findings indicate that Bio-C Temp is biocompatible and had no hepatotoxicity effect.

## Introduction

The complexity of the root canal system can interfere with its complete cleaning and disinfection and even after biomechanical preparation, microorganisms can remain in the root canal system, leading to endodontic treatment failure^1^. Therefore, the use of intracanal medication may enhance the disinfection of root canal system, apical cementum and dentinal tubules^2^. Moreover, intracanal medication acts as a physical barrier preventing reinfection and reducing the risk of bacteria proliferation^3^.

The calcium hydroxide pastes have been widely used as an intracanal medication acting as a complement to biomechanical preparation due to its antimicrobial potential^2^, ability to dissolve organic tissue^4^, stimulate the osteoblast proliferation^5^, and induction of mineralized tissue formation^6^. Despite these good properties, the calcium hydroxide-based pastes have low radiopacity and flow capacity, which difficult their insertion into the root canal^7–9^. Moreover, the use of intracanal calcium hydroxide medication for prolonged period, such as for 6 months^10^, induces collagen degradation and, hence, weakening the root dentine^11^ and increasing the risk of fracture^12^. Thus, there is the search for new medications or associations with the materials routinely used in order to enhance the properties of the intracanal dressing^13^.

The bioceramic material has been used due to excellent biocompatibility and bioactive potential of the calcium silicate^14^ which promotes the deposition of hydroxyapatite on its surface^15^. The bioactivity of bioceramic materials favor osteoblasts survival and differentiation, cells actively involved in the periapical repair^16^ enabling the formation of mineralized tissue^17^. Thus, there is currently a growing interest in the development of new materials based on biocompatibility and bioactivity of calcium silicate^18^.

The Angelus (Angelus, Londrina, PR, Brazil) has developed a bioceramic intracanal medication, Bio-C Temp, ready to use. The Bio-C Temp is indicated as an intracanal dressing^18^ for endodontic treatment or retreatment, pulpotomy and treatment of immature permanent teeth to induce the apexification^19^. According to the manufacturer, Bio-C Temp contains calcium silicates associated with calcium tungstate and titanium oxide radiopacifiers, besides calcium aluminate, calcium oxide and base resin^20^.

The Bio-C Temp medication has shown acceptable cell viability when diluted extracts of this medication are added to immortalized fibroblasts culture^18^, human dental pulp cells (hDPCs)^21^ and osteoblast-like cells (Saos-2)^19^. The cells from Saos-2 co-cultured with Bio-C Temp extracts exhibited alkaline phosphatase activity and red alizarin-positive mineralized nodules, suggesting that this bioceramic medication exerts an osteogenic activity. The osteogenic activity induced by Bio-C Temp on Saos-2 was similar to that seen in Saos-2 cultures with extracts of calcium hydroxide-based medicaments^19^. Moreover, this bioceramic intracanal medication provides an alkaline pH (pH around 10.79 at 7 days) and releases calcium to the microenvironment^18^, supporting the concept that Bio-C Temp has bioactive potential^19^. Bio-c Temp exhibits greater radiopacity (about 7 mm/Al) than Ultracal XS (Ultradent Products, South Jordan, UT, USA), a calcium hydroxide-based paste^18^.

Despite the physicochemical and in vitro studies, there are no in vivo studies on the biological properties of this material. The implantation of polyethylene tubes filled with endodontic materials into subcutaneous tissues of rats is a method widely used to evaluate the tissue response to the materials as well as the type and extent of the inflammatory reaction^22–25^. Sections of implants surrounded by connective tissue embedded in paraffin allows us to investigate the complex cascade of cellular and molecular events involved in the tissue response by morphological and immunohistochemical analyses^23–25^.

This study aimed to evaluate whether Bio-C Temp induces changes in the serum glutamic-oxaloacetic transaminase (GOT) and glutamic-pyruvic transaminase (GPT) levels and the tissue reaction induced by this bioceramic intracanal medication in the subcutaneous connective tissue in different time points. The biological response induced by Bio-C Temp was compared with a calcium hydroxide-based paste (Calen, SS.White, Rio de Janeiro, RJ, Brazil), used as gold pattern. The null hypothesis was that Bio-C Temp does not present better biological properties when compared with the calcium hydroxide paste.

## Results

### Serum hepatic enzymes level

**Figure 1:**
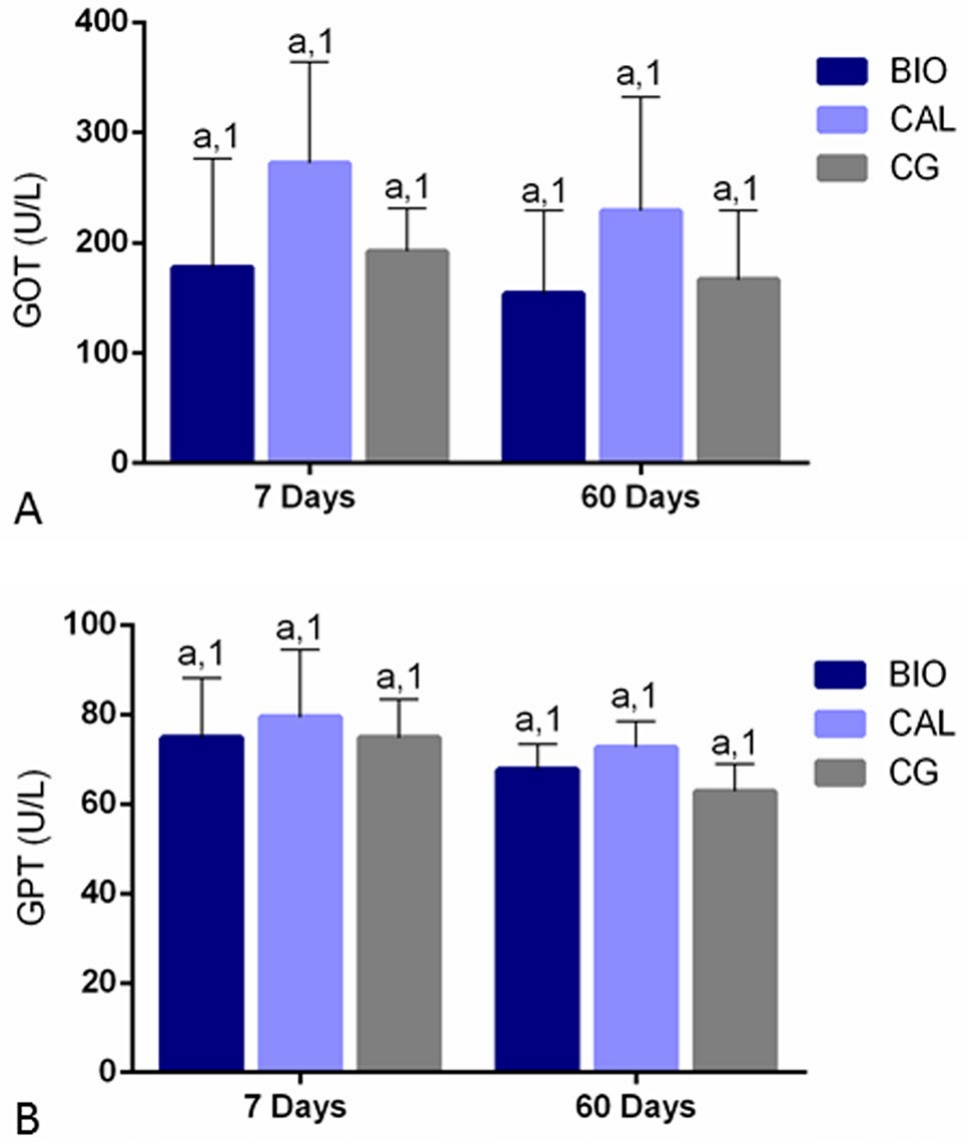
(**A**,**B**) The serum levels of glutamic-oxaloacetic transaminase (GOT—**A**) and glutamic-pyruvic transaminase (GPT—**B**) in Bio-C Temp, Calen and Control groups at 7 and 60 days. In rats with intracanal medication, the GOT and GPT levels were similar to CG rats. From 7 to 60 days, significant differences are not seen in the serum GOT and GPT levels. Values expressed as mean ± SD. Tukey’s test (p ≤ 0.05). In each period, superscript letters indicate the analysis among groups; same letters = no significant difference. The superscript numbers indicate the analysis of each group over time; same numbers = no significant difference.

As shown in the Fig. 1A,B significant differences in serum GOT and GPT concentrations among BIO, CAL and CG specimens (p > 0.05) were not observed, at 7 and 60 days. Moreover, from 7 to 60 days, no significant difference was also seen in serum GOT and GPT levels in all groups (p > 0.05).

### Morphological findings, capsule thickness and numerical density of number of inflammatory cells

**Figure 2:**
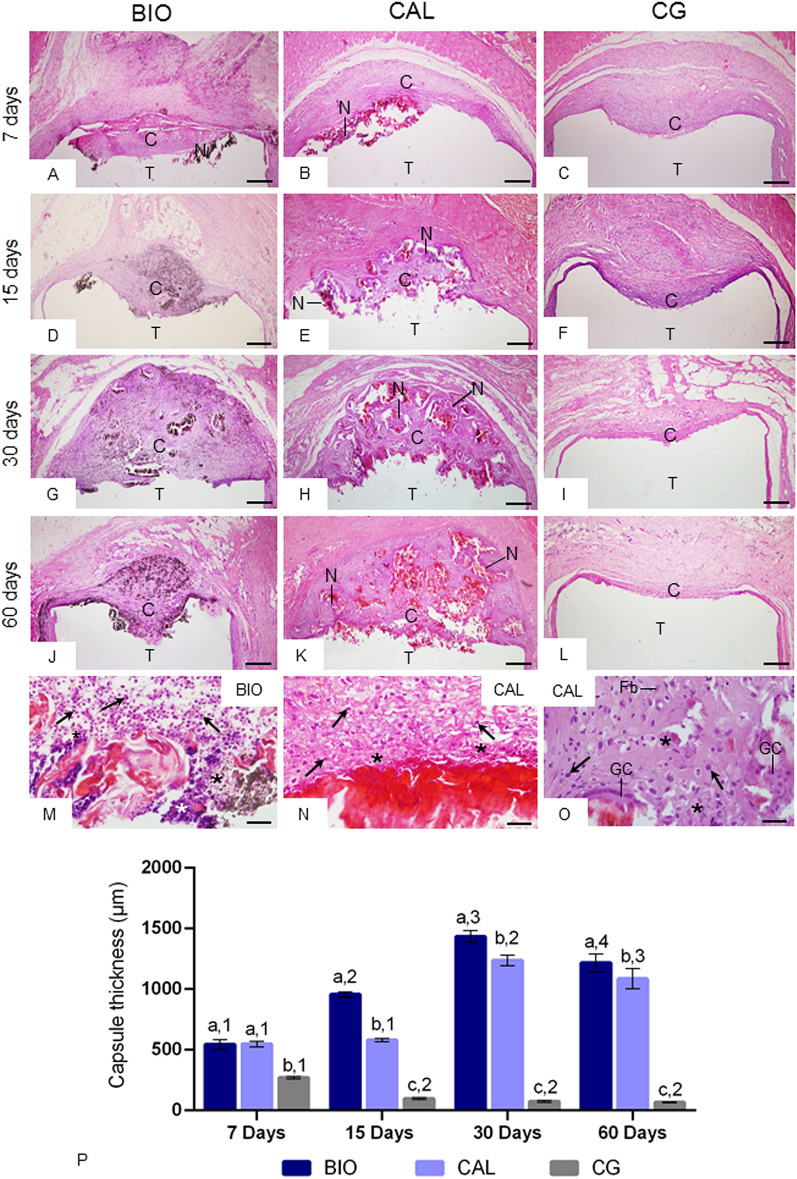
(**A**–**L**) Light micrographs of sections showing an overview of capsules adjacent to the opening of the implanted tubes (T). In all periods, well-defined capsules (C) are seen. In BIO specimens, several particles (brown/black color) are dispersed throughout the capsules (C). In all periods, thin capsules are observed in the CG. Note necrosis areas (N) in the capsules around Bio-C Temp (**A**) and Calen paste (**B**,**E**,**H**,**K**). Bars: 152 µm. (**M**–**O**) High magnification of portions of capsules in close juxtaposition to the Bio-C Temp at 7 days (**M**) and Calen paste at 7 (**N**) and 60 (**O**) days; necrosis areas (asterisks) are observed. Arrows, inflammatory cells; Fb, fibroblast; GC, multinucleated giant cell. Bars: 27 µm. HE. (**P**) The graphic shows the values (expressed as mean ± standard deviation) of capsule thickness (in µm). In each period, the comparison among the groups is indicated by superscript letters; different letters = significant difference. The superscript numbers indicate the analysis of each group over time; different numbers = significant difference. Tukey’s test (p ≤ 0.05).

In all groups, well-defined capsules were around the implants (Fig. 2A–L). In BIO and CAL there was an increase in the thickness of the capsules over time. Moreover, material particles were often observed dispersed by the capsules (Fig. 2A,B,D,E,G,H,J,K). At 7 days, necrotic areas were observed in the surface of capsules of BIO and CAL (Fig. 2A,B); in the CAL, necrotic areas were seen at all periods (Fig. 2B,E,H,K,N,O). Necrotic areas were in close juxtaposition to the medications. At initial periods, several inflammatory cells and scarce extracellular matrix components were next to the necrotic areas in BIO (Fig. 2M) and CAL (Fig. 2N) specimens. At 60 days, necrotic areas were seen in the capsules adjacent to the Calen medication (Fig. 2O). The CG specimens were surrounded by thin capsules (Fig. 2C,F,I,L).

According to Fig. 2P, no significant difference was found in the capsule thickness between BIO and CAL specimens at 7 days (p = 0.98). The capsules of BIO specimens were significantly thicker than in CAL (p < 0.0001) at 15, 30 and 60 days. In BIO and CAL specimens, a significant increase in the capsule thickness was verified at 30 days, but a significant reduction was noticed at 60 days (p < 0.0001). At all periods, CG specimens exhibited the lowest values in the capsule thickness and a significant reduction was detected over time (p < 0.0001).

**Figure 3:**
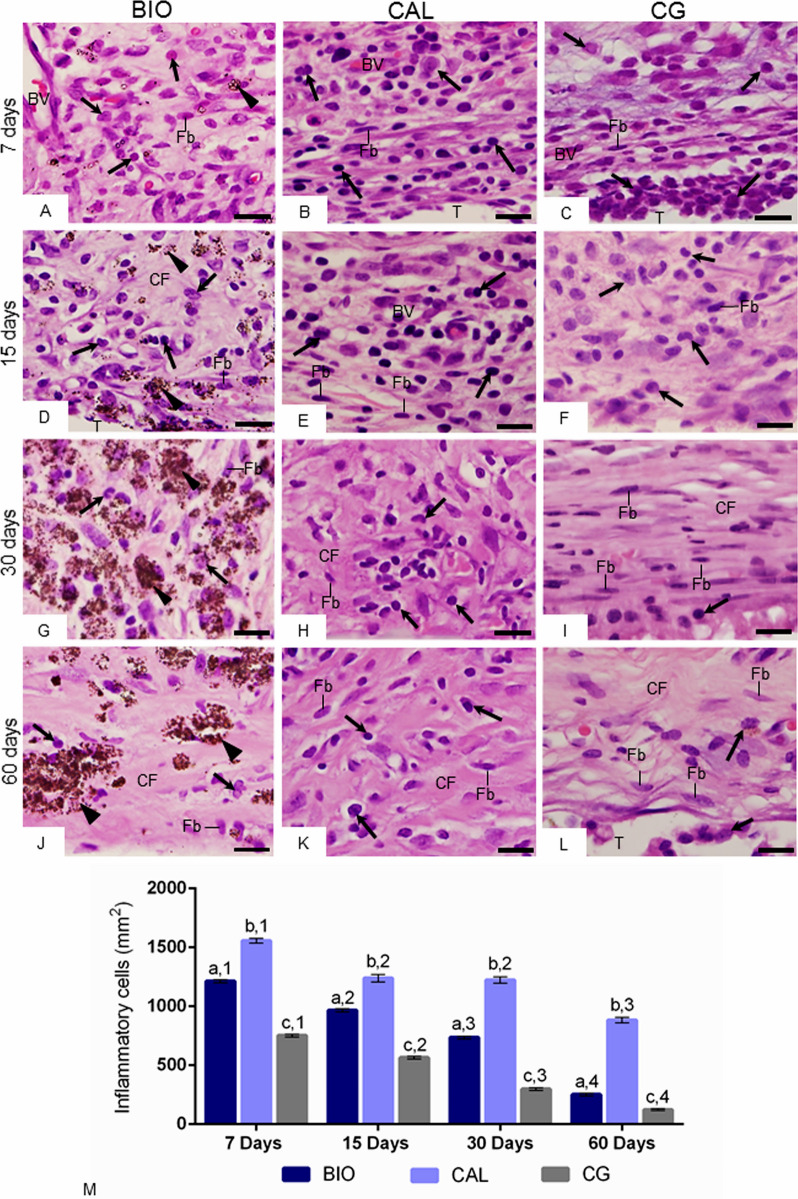
(**A**–**L**) Light micrographs showing the innermost portion of capsules. Inflammatory cells (arrows), fibroblasts (Fb), collagen fibers (CF) and material particles (arrowheads) are seen in the capsules. At 15, 30 and 60 days, the capsules of the control group exhibit few inflammatory cells (arrows) and a gradual increase in fibroblasts (Fb) and collagen fibers (CF). BV, blood vessels; HE. Bars: 18 µm. (**M**) The graphic shows the values (expressed as mean ± standard deviation) of the numerical density of inflammatory cells in the capsules. In each period, the comparison among the groups is indicated by superscript letters; different letters = significant difference. The superscript numbers indicate the analysis of each group over time; different numbers = significant difference. Tukey’s test (p ≤ 0.05).

At 7 days, the capsules of all groups contained several inflammatory cells, mainly macrophages and lymphocytes, and few fibroblasts (Fig. 3A–C). However, significant differences were observed among the groups (p < 0.0001); the lowest values of inflammatory cells were obtained in CG capsules while the highest values were found in CAL specimens (Fig. 3A–M). In BIO and CG, a significant reduction in the number of inflammatory cells was observed over time (p < 0.0001). Although the number of inflammatory cells reduced significantly in CAL specimens from 7 to 15 days (p < 0.0001), no significant difference (p = 0.61) was detected between the periods of 15 and 30 days. At 60 days, the number of inflammatory cells in all groups was lower (p < 0.0001) than other time points (Fig. 3M).

### Immunohistochemical detection of IL-6

**Figure 4:**
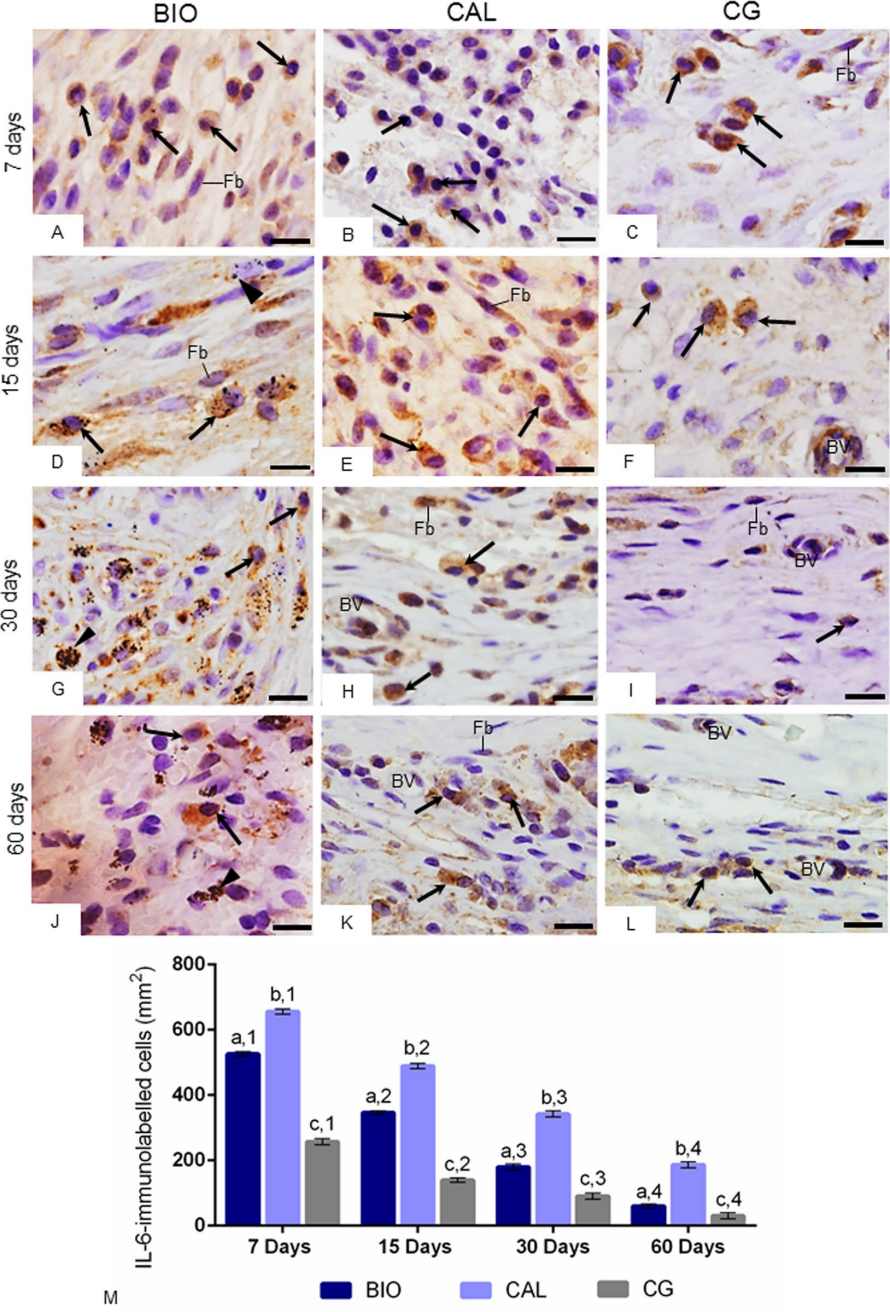
(**A**–**L**) Light micrographs showing portions of capsule of sections submitted to immunohistochemistry reaction for detection of IL-6 (brown/yellow color) and counterstained with hematoxylin. In all periods, inflammatory cells with strong immunolabelling in their cytoplasm (arrows) are present in the capsules of all groups. Immunolabelled fibroblasts (Fb) are also observed. BV, blood vessels; material particles (arrowheads). Bars: 18 µm. (**M**) The graphic shows the values (expressed as mean ± standard deviation) of the numerical density of IL-6-immunolabelled cells in the capsules. In each period, the comparison among the groups is indicated by superscript letters; different letters = significant difference. The superscript numbers indicate the analysis of each group over time; different numbers = significant difference. Tukey’s test (p ≤ 0.05).

The capsules of all groups showed inflammatory cells, particularly neutrophils and macrophages, containing strong immunolabelling in their cytoplasm (brown-yellow color) in all periods. Moreover, some immunolabelled fibroblasts were also observed (Fig. 4A–L). A reduced immunoexpression was observed in the capsules of CG, particularly at 30 and 60 days (Fig. 4C,F,I,L).

The quantitative analysis (Fig. 4M) revealed that the number of IL-6-immunostained cells decreased significantly from 7 to 60 days (p < 0.0001), in all groups. In all periods, the number of immunolabelled cells was significantly lower around the BIO specimens than in CAL (p < 0.0001) while the lowest values were observed in the capsules of CG.

### Immunohistochemical detection of IL-10

**Figure 5:**
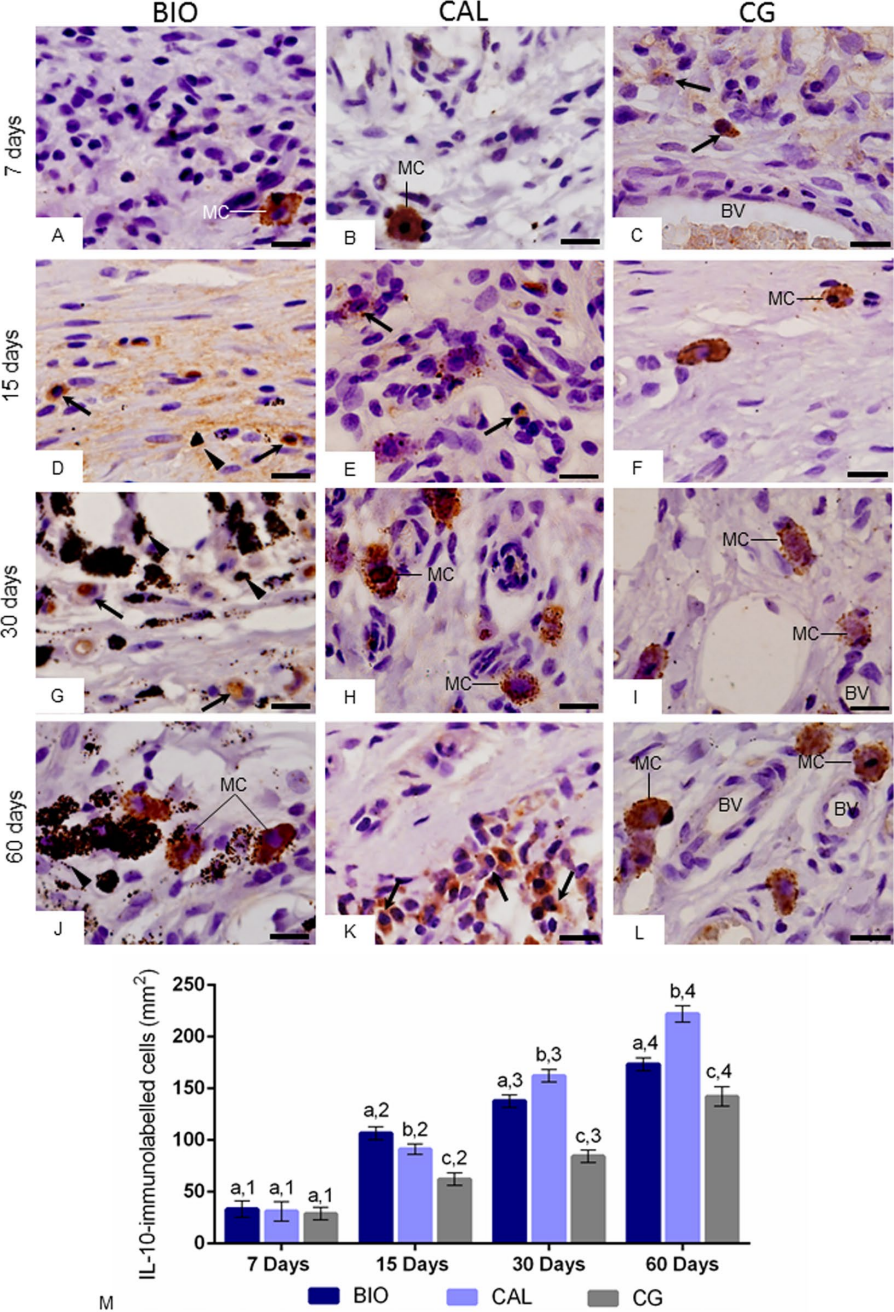
(**A**–**L**) Light micrographs showing portions of capsule of sections submitted to immunohistochemistry for detection of IL-10 (brown/yellow color) and counterstained with hematoxylin. Note that few immunostained cells are present in the capsules of all groups, particularly at 7 days (**A**–**C**). In all groups, an evident immunolabelling is present in the mast cells (MC). Arrows, inflammatory cells; BV, blood vessels; material particles, arrowheads. Bars: 18 µm. (**M**) The graphic shows the values (expressed as mean ± standard deviation) of the numerical density of IL-10-immunolabelled cells in the capsules. In each period, the comparison among the groups is indicated by superscript letters; different letters = significant difference. The superscript numbers indicate the analysis of each group over time; different numbers = significant difference. Tukey’s test (p ≤ 0.05).

In all periods, the immunohistochemistry reaction for detection of IL-10 revealed immunolabelled cells (brown-yellow color) in the capsules of all groups. Although some inflammatory cells and fibroblasts exhibited immunolabelling for IL-10, strong immunolabelling in the cytoplasm of mast cells was often seen in the capsules of all groups (Fig. 5A–L).

According to Fig. 5M, the lowest values of IL-10-immunolabelled cells were found in all groups at 7 days. From 7 to 60 days, the immunoexpression of IL-10 increased significantly in all groups (p < 0.0001). At 7 days, no significant difference among the groups was detected (p > 0.05). At 15, 30 and 60 days, the number of IL-10-immunolabelled cells was significantly lower in CG specimens than in BIO and CAL groups (p < 0.0001). On 15th day, the highest values were found in BIO specimens while, at 30 and 60 days, the highest values were observed in the CAL specimens (p < 0.0001).

### Birefringent collagen content in capsules

**Figure 6:**
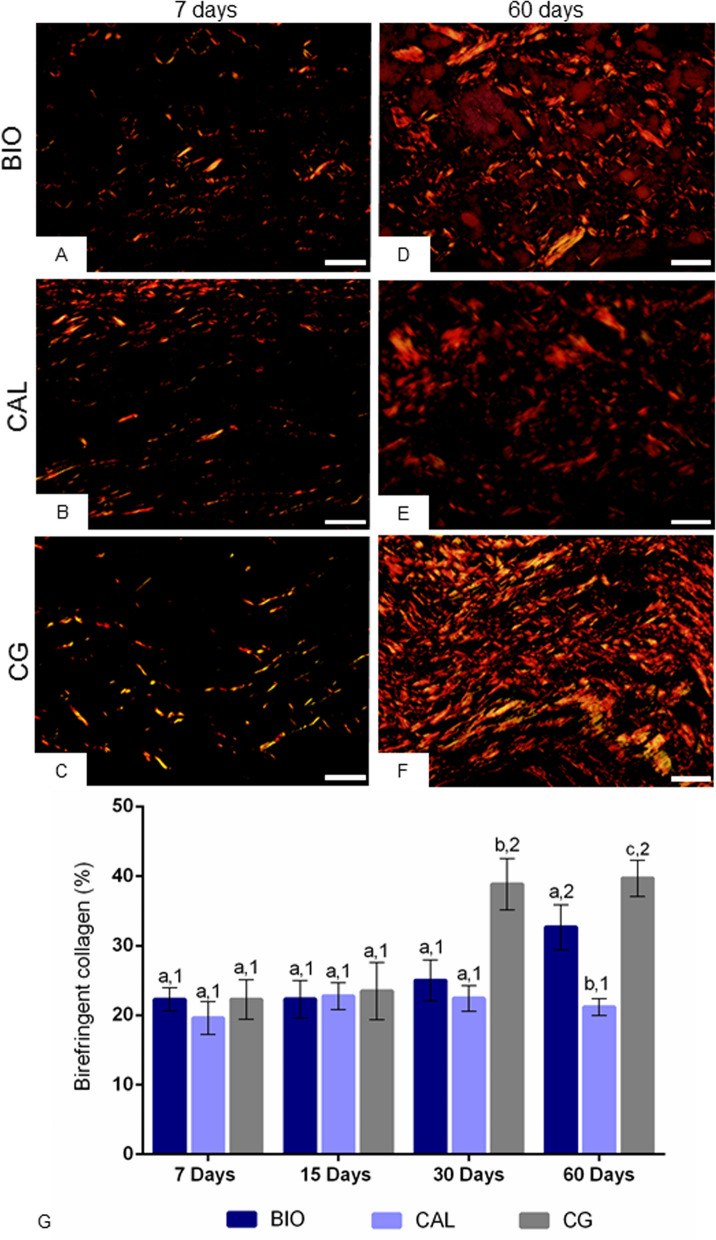
(**A**–**F**) Light micrographs showing portions of capsule from sections subjected to picrosirius-red and analyzed under polarized illumination. At 7 days, thin birefringent collagen fibers (in red-orange) are dispersed in the capsules. At 60 days, the capsules exhibit thick bundles of birefringent collagen; note an accentuated birefringence in capsules of BIO (**D**) and CG (**F**) groups compared to the CAL group (**E**). Bars: 18 µm. (**G**) The graphic shows the values (expressed as mean ± standard deviation) of the amount of birefringent collagen (in percentage) in the capsules. In each period, the comparison among the groups is indicated by superscript letters; different letters = significant difference. The superscript numbers indicate the analysis of each group over time; different numbers = significant difference. Tukey’s test (p ≤ 0.05).

At 7 days, all groups exhibited capsules with few and thin birefringent collagen fibers in yellow/red color (Fig. 6A–C). On 60th day, an evident increase in the birefringence was observed in the capsules of all groups. In this period, an increase in the bundles of collagen fibers exhibiting red/orange birefringence was seen in the capsules around all implants (Fig. 6D–F). The quantitative analysis (Fig. 6G) showed that the amount of birefringent collagen was similar among the groups (p ˃ 0.05) at 7 and 15 days. Although no significant difference between BIO and CAL was found at 30 days, the amount of birefringent collagen was significantly greater in BIO than CAL specimens at 60 days (p < 0.0001). After 30 and 60 days, the collagen content was significantly greater in CG than in BIO and CAL groups (p < 0.0001). The analysis revealed a significant increase in the collagen amount in BIO group at 60 days (p < 0.0001). In contrast, no significant difference was detected in the CAL group over time (p > 0.05). In the CG, the amount of collagen was significantly greater at 30 and 60 days than in the periods of 7 and 15 days.

## Discussion

This is the first study that evaluated the serum GOT and GPT levels and biocompatibility in vivo of Bio-C Temp bioceramic intracanal medicament after implantation into the subcutaneous tissues of rats. Morphological and quantitative analyses of capsules around intracanal medications at different time points allow evaluating the injured tissue as well as the complex cascade of cytokines caused by these medicaments, which may lead to chronic inflammatory reaction or tissue repair^26–28^. Furthermore, this methodology allows us to investigate whether dental materials could promote systemic changes^28^. The null hypothesis was rejected since Bio-C Temp favouring the connective tissue repair quickly in comparison with the calcium hydroxide paste.

In the present study, bioceramic medication and calcium hydroxide-based paste did not promote changes in serum GOT and GPT levels in comparison with CG after 7 and 60 days of implantation. Therefore, the intracanal medications may not be hepatotoxic, since the measurement in serum of GOT and GPT levels can be used as biochemical parameters of liver function^29^. Usually, hepatic enzymatic changes are associated with structural liver damage^30^. Cellular viability hDPCs^21^ and Saos-2^19^ is directly correlated with the concentration of Bio-C Temp extracts. The cytotoxicity of this bioceramic intracanal medication when added in high concentrations has been associated with the titanium dioxide^21^, which promotes cell damage culminating in cell death by apoptosis^31^. According to the manufacturer, Bio-C Temp also contains base resin in its composition^20^. It is known that resins can release substances, which may promote local and systemic adverse reactions^32^. Here, serum GOT and GPT levels suggest that Bio-C Temp and calcium hydroxide-based pastes may not promote liver injury. However, further studies are need to evaluate whether these intracanal medications promote structural changes in the liver.

The morphological and quantitative analyses revealed that, in all groups, the greatest values of inflammatory cells were observed at 7 days. The inflammatory reaction induced by empty polyethylene tubes suggests that surgical trauma may stimulate the recruitment of inflammatory cells and, may be responsible, at least the part, for highest numerical density of inflammatory cells initially found, as reported in other studies^22,26,27,33,34^. However, the number of inflammatory cells in the BIO and CAL specimens was around two folds greater than in CG, which may be attributed to the alkaline pH and chemical composition of these intracanal medications. The Calen paste provides an alkaline pH around 12.4 to microenvironment^35^ and, Bio-C Temp provides pH around 10.79 after 7 days^18^. Although calcium hydroxide is not present in the composition of Bio-C Temp, it is known that calcium silicate-based materials react with water raising hydroxyl and calcium ions, resulting in the calcium hydroxide deposition^18^.

The alkaline pH induces the recruitment of inflammatory cells to the microenvironment^25,26,36^ and formation of a coagulation necrosis zone^36^ in the tissue surface in contact with the calcium hydroxide-based materials^9,37,38^. The necrosis process observed in the initial period in the capsules of CAL and BIO is caused by ions calcium and hydroxyl released by these medications when in contact with tissue fluids^39,40^. Considering that in CAL specimens, the necrotic areas remained until 60 days and the greatest number of inflammatory cells was observed, it is conceivable to suggest that the release of hydroxyl may be maintained for a long time in the Calen paste. Superficial necrosis areas may be explained due to high pH provided by medications that induces the rupture of cellular membranes and their content leak out, culminating in an inflammatory reaction^9,25^. However, the cellular damage promoted by these medications may be restrict to tissue area in close juxtaposition to the medication^38^. The alkaline pH promotes vascular proliferation and stimulates the recruitment the macrophages, which migrate to these sites, phagocytizing cell debris and extracellular matrix components. Moreover, superficial necrosis stimulates proliferation and differentiation of mesenchymal cells culminating with collagen formation^25,39^. Calcium ions released by intracanal medications contribute to precipitation of calcium carbonate on injured areas culminating in the mineralization of newly formed collagen^41^.

In the present study, capsules around Calen paste specimens exhibited greater number of IL-6-immunostained cells and lower amount of red/orange birefringent collagen fibers than in Bio-C Temp specimens. Since red/orange birefringent collagen fibers are frequently associated with type I collagen^42,43^, this finding indicates a different collagen deposition pattern stimulated by Bio-C Temp and Calen medications. The Bio-C Temp allowed a marked formation of thick collagen fiber bundles in comparison to the Calen. Moreover, the intense IL-6-immunoexpression point to a harmful effect of Calen on the connective tissue since IL-6 is a multifunctional pro-inflammatory cytokine, being considered an indicative parameter of the inflammatory reaction intensity^24,44^. Studies have shown a direct correlation between the reduction in the number of inflammatory cells and IL-6 immunoexpression in the capsules adjacent to the calcium silicate-based materials implanted in rat subcutaneous^23,24,27,45^. In the present study, the reduction in the immunoexpression of IL-6 was also accompanied by decrease in the number of inflammatory cells reinforcing the concept that this interleukin may modulate the inflammatory reaction in response to the endodontic materials. The irritating potential of Calen can also be justified by the harmful activity of zinc ions from the radiopacifying agent zinc oxide present in this paste^46^. However, this irritant potential decreases over time corroborating with other studies^9,37,38^.

The thickness of capsules around BIO and CAL specimens increased significantly until on 30th day. The thickening of connective tissue around intracanal medications was accompanied by increase of material particles dispersed throughout the capsules. The massive presence of material particles may be due to the flow and solubility of these pastes, particularly, of Bio-C Temp. However, these properties are needed to the filling of the root canals and penetration into the dentinal tubules^3^, acting in these areas, which are not achieved by the channel instrumentation^2^. The Calen has as vehicle the polyethylene glycol 400, which improves the dissolution of calcium hydroxide and release of hydroxyl^35^. Bio-C Temp contains in its composition the base resin as a vehicle, which allows better insertion of the material in the root canals^47^. From 30 to 60 days, the thickness of capsules around intracanal medications reduced significantly suggesting a remodelling process of the connective tissue of these capsules.

There is evidence showing a strong positive correlation between IL-6 and matrix metalloproteinases (MMP), such as MMP-1 and MMP-9, enzymes responsible for extracellular matrix degradation^48^. Here, it is possible to suggest that the IL-6 may exert a control on the degradation of extracellular matrix components since the highest values of this interleukin were parallel to lowest collagen content in the CAL specimens while the lowest immunoexpression of IL-6 was concomitant with accentuated amount of collagen in CG specimens. These findings point to a participation of IL-6 in the breakdown of extracellular matrix components in the capsules around implants in the subcutaneous tissue.

Moreover, the reduction in the immunoexpression of IL-6 was also accompanied by gradual increase of IL-10-immunostaining over time. In the present study, several mast cells exhibited an accentuated immunolabelling for IL-10. Although IL-10-immunopositive mast cells were observed in all periods, the marked presence of immunostained mast cells at 60 days may be associated with the intense collagen formation, suggesting tissue repair. One of the anti-inflammatory actions of IL-10 is the ability to modulate the production of inflammatory cytokines by mast cells. Mast cells contain IL-10 receptors, and, via IL-10, release several inflammatory mediators, which mediate the immune and inflammatory responses^49^ and participate in the tissue repair and remodelling^44,49^. The accentuated number of mast cells in the capsules around a reparative calcium silicate-based biomaterial, Biodentine (Septodont, Saint-Maur-des-Fossèes, França), was associated with fibroblasts proliferation and, consequently, with the collagen formation supporting the concept that mast cells are involved in the connective tissue repair^44^. Moreover, there is evidence that IL-10 inhibits IL-6 production by mast cells in response to inflammation and bacterial infection^50^. Thus, our findings taken together indicate that the increase in the IL-10 may inhibit the immunoexpression of IL-6 in the capsules mitigating the inflammatory reaction promoted by bioceramic and calcium hydroxide intracanal medications implanted in the subcutaneous tissues.

No study evaluating the biological in vivo behavior of the Bio-C Temp was found in the literature. Our findings indicate that, in the initial period, bioceramic medication induced an intense inflammatory reaction, which gradually reduced over time. Thus, at 60 days, the capsules around Bio-C Temp specimens showed bundles of collagen fibers surrounding the material particles, pointing to a connective tissue reorganization, and, therefore, indicating that this bioceramic medication is biocompatible. The biocompatibility of tricalcium silicate-based materials^26,33,34^ seems to be due to the stable chemical comportment of the calcium silicate in biological environment^51^. Therefore, it is expected that Bio-C Temp, when in contact with periapical tissues, may induce the tissue repair. Although subcutaneous implantation in rats is a methodology recommended by ISO 109933^52^ to assess the biocompatibility of dental materials, it is important to emphasize that the intracanal medication has contact with infected and/or inflamed periradicular tissues. Moreover, the periodontal microenvironment contains, differently from subcutaneous connective tissue, other cell types besides fibroblasts, such as cementoblasts, osteoblasts and osteoclasts. Despite its biocompatibility, it is still unclear whether Bio-C Temp has antimicrobial efficacy and whether possible residues of this medication might interfere in the properties of endodontic sealers. Therefore, further studies are necessary to confirm the safety and viability of this bioceramic intracanal medication in clinical practice.

In conclusion, serum GOT and GPT levels suggest that bioceramic and calcium hydroxide-based intracanal medications had no hepatotoxicity effect. Bio-C Temp caused initial tissue damage that was quickly suppressed in comparison to those caused by calcium hydroxide-based paste, favouring the repair of connective tissue indicating, therefore, that this bioceramic material is biocompatible.

## Materials and methods

### Experiment design

The present research protocol was approved by the Ethical Committee for Animal Research of Araraquara Dental School (FOAr-Araraquara, UNESP, São Paulo, Brazil; CEUA # 22/2018). Sixty adult male Holtzman rats (*Rattus norvegicus albinus*) weighing 220–250 g were used. The rats were maintained in polyethylene cages under 12 h light/12 h dark cycle at controlled temperature (23 ± 2 °C) and humidity (55 ± 10%), with water and food provided ad libitum. The study was carried out in accordance with the US National Institute of Health Guide for the Care and Use of Laboratory Animals (NIH Publications No. 80-23, 1996). The experiment and analyses method were conducted in accordance with ARRIVE guidelines 2.0 (Animal Research: Reporting of In Vivo Experiments).

**Figure 7:**
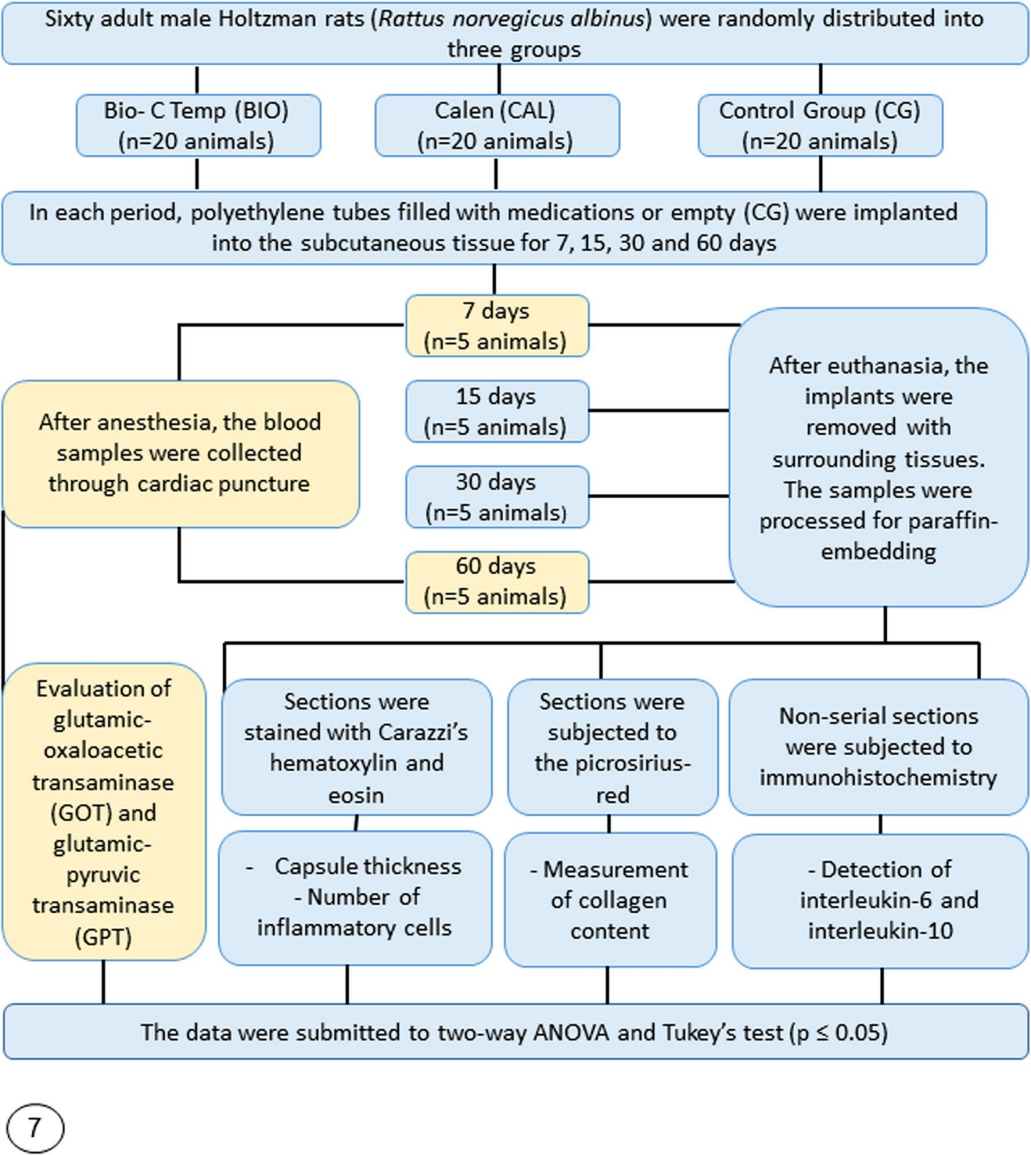
Flowchart of experimental design with total number of animals, number of animals per group and methodological analyses performed in the study.

Sixty rats were randomly distributed into three groups containing 20 animals each (Fig. 7): BIO (BIO-C TEMP Group, Angelus, Londrina, Brazil); CAL (Calen Group, SS. White Art. Dent. Ltda, RJ, Brazil) and CG (control group, empty polyethylene tubes). Each cage was identified according to the group and period. The sample size for this study was calculated based in previous studies^24,25,28^. The size sample was calculated considering an alpha error of 0.05 to recognize a significant difference and 90% test power for detection of 50% difference among experimental groups and CG. Thus, a sample of 5 rats per group in each time point was required, totalling twenty animals per group.

The animals were anaesthetized with ketamine hydrochloride (80 mg/kg of body weight) and xylazine hydrochloride (8 mg/kg of body weight) by intraperitoneal route. After shaved and disinfection with 5% iodine solution, a 2.0 cm-long incision was made in a head-to-tail orientation using a no. 15 scalpel (Fibra Cirúrgica, Joinvile,SC, Brazil). The polyethylene tube (Embramed Indústria e Comércio Ltda, São Paulo, São Paulo, Brazil) with 10.0 mm length and 1.6 mm diameter, previously sterilized with ethylene oxide, were implanted into the subcutaneous tissue. In each animal, two polyethylene tubes of the same group were implanted for 7, 15, 30 and 60 days (Fig. 7).

### Serum hepatic enzymes level

To evaluate whether intracanal medications cause changes in the hepatic enzymes (GOT and GPT), the blood samples were collected through cardiac puncture with BD Vacutainer^®^ Blood Collection Tubes (SSTII Plus, BD Biosciences) at 7 and 60 days (Fig. 7). After clot formation, the blood was centrifuged (Excelsa^®^ II 206 BL; Fanem Ltda., Guarulhos, SP, Brazil) at 3500 rpm for 10 min and the serum was stored at -20° C. The GOT and GPT serum concentrations were determined using the AST (aspartate aminotransferase) and ALT (Alanine Aminotransferase), respectively, by chemiluminescence immunoassay kits (Beckman Coulter, CAR, USA). Concentrations were measured using a multiparametric automatic analyzer (Cobas ÍNTEGRA^®^ 400 Plus; Roche Diagnóstica Brasil Ltda., São Paulo, SP, Brazil). The analyses were performed at São Lucas Clinical and Microbiological Laboratory. The experiments were carried out in duplicate and the averages were calculated.

### Histological processing of samples

The tissue reaction promoted by intracanal medications was carried out in all groups at 7, 15, 30, and 60 days. The animals were euthanized with overdose of ketamine and xylazine and, subsequently, the specimens containing the implants surrounded by tissues were removed and immersed in 4% formaldehyde (freshly prepared from paraformaldehyde) buffered at pH 7.2 with 0.1 M sodium phosphate. After 48 h, the specimens were dehydrated with ethanol graded concentrations, treated with xylene, and embedded in paraffin. In each specimen, forty serial longitudinal sections (6 µm thick) were obtained and adhered to glass slides. Three non-serial sections were stained with Carazzi’s hematoxylin and eosin (HE) for morphological analysis of the capsules, and to estimate the capsule thickness and the numerical density of inflammatory cells. Three non-serial sections were subjected to the picrosirius-red to estimate the collagen content. Other non-serial sections were adhered to slides previously treated with silane 4% (Sigma-Aldrich) and submitted to the immunohistochemistry reactions for detection of interleukin-6 (IL-6) and IL-10 (Fig. 7).

The histological description and quantitative data were obtained using a digital camera (DP-71, Olympus, Tokyo, Japan) attached to a light microscope (Olympus BX-51, Tokyo, Japan) and an image analysis system (Image-Pro Express 6.0, Olympus). The analyses were conducted by one calibrated and blinded examiner.

### Numerical density of inflammatory cells

The number of inflammatory cells was obtained from three non-serial sections of each implant. In each section, an image of the central portion of capsule in close juxtaposition to the opening of implanted tube was captured at × 695 magnification (objective lens: × 40). In this standardized field (0.09 mm^2^), the number of inflammatory cells (neutrophils, lymphocytes, plasma cells, and macrophages) was computed using the image analysis system. Thus, the number of inflammatory cells per millimetre square of capsule was obtained dividing the total number of inflammatory cells by the total standardized field of capsule^24,26,27,33,45^.

### Capsule thickness

For each specimen, three HE-stained non-serial sections were used, totalling15 sections per group in each period. To estimate the capsule thickness, an image at × 65 magnification was captured (objective lens: × 4); the measurement was made from the capsule surface to the adjacent tissues (in micrometers). In each specimen, the mean value of capsule thickness was calculated from the three sections analyzed^23,24,45^.

### Immunohistochemical detection of IL-6 and IL-10

For the detection of interleukin-6 and interleukin-10, mouse monoclonal anti-IL-6 antibody (Abcam Inc., Cambridge, MA, USA; code: ab 9324) and mouse monoclonal anti-IL-10 antibody (Santa Cruz Biotechnology, Santa Cruz, CA, USA; code SC-8438) were used. After deparaffinization and hydration, the slides were immersed in 0.001 M sodium citrate buffer (pH 6.0) and subjected to microwave for 20 min at 96–98 °C. After cooling-off, the slides were washed in 0.01 M sodium phosphate buffer (PBS) for 15 min. For inactivation of endogenous peroxidase, the sections were immersed for 30 min in 5% aqueous hydrogen peroxide. After washing in PBS, the sections were incubated for 20 min with 2% bovine serum albumin (Sigma-Aldrich Co., Saint Louis, Missouri, USA) at room temperature. Afterwards, the sections were incubated with anti-IL-6 antibody (diluted at 1:400) or anti-IL-10 antibody (diluted at 1:100) in a humid chamber at 4 °C for 16 h. After washings with PBS, the sections were incubated for 60 min with the Labeled StreptAvidin-Biotin kit (Universal Dako LSAB, Dako Inc., Carpinteria, CA, USA; K0675) at room temperature. Peroxidase activity was revealed by 3,3′-diaminobenzidine chromogen (DAB substrate, Vector Laboratories, Burlingame, CA, USA) for 3 min. The sections were stained with Carazzi's hematoxylin. As a negative control, the sections were incubated with non-immune serum. Subsequently, the quantification of immunostained cells was performed.

The number of IL6- and IL10-immunolabelled cells was estimated in the capsules of five specimens of each group/period. In each section, a standardized area (0.09 mm^2^) was captured at × 695 magnification (objective lens: × 40). Using the image analysis system, the number of immunolabelled cells (brown-yellow color) was computed^24,27,33,45^.

### Measurement of birefringent collagen

The amount of collagen was estimated in three non-serial sections stained with picrosirius-red and analyzed under polarized illumination. In each section, the birefringent collagen was measured in a standardized field (0.09 mm^2^) of the capsule at × 695 magnification (objective lens: × 40). All images were captured with standardized light intensity, field diaphragm aperture, condenser diaphragm and exposure time. The amount of birefringent collagen was performed using the ImageJ software, which provided the number of pixels of each color frequency (red, orange, yellow and green) occupied in the total pixel value of the captured images^23,34^.

### Statistical analysis

The GraphPad Prism 6.01 program (GraphPad Software, Inc., La Jolla, CA, USA) was used, and the data were submitted to two-way ANOVA analysis of variance, followed by the Tukey test (p ≤ 0.05). All data were presented as mean ± standard deviation.
